# Neural Substrate Expansion for the Restoration of Brain Function

**DOI:** 10.3389/fnsys.2016.00001

**Published:** 2016-01-25

**Authors:** H. Isaac Chen, Dennis Jgamadze, Mijail D. Serruya, D. Kacy Cullen, John A. Wolf, Douglas H. Smith

**Affiliations:** ^1^Department of Neurosurgery, University of PennsylvaniaPhiladelphia, PA, USA; ^2^Philadelphia Veterans Affairs Medical CenterPhiladelphia, PA, USA; ^3^Department of Neurology, Thomas Jefferson UniversityPhiladelphia, PA, USA

**Keywords:** axons, brain repair, neural interfaces, neuronal networks, stem cells, tissue engineering

## Abstract

Restoring neurological and cognitive function in individuals who have suffered brain damage is one of the principal objectives of modern translational neuroscience. Electrical stimulation approaches, such as deep-brain stimulation, have achieved the most clinical success, but they ultimately may be limited by the computational capacity of the residual cerebral circuitry. An alternative strategy is brain substrate expansion, in which the computational capacity of the brain is augmented through the addition of new processing units and the reconstitution of network connectivity. This latter approach has been explored to some degree using both biological and electronic means but thus far has not demonstrated the ability to reestablish the function of large-scale neuronal networks. In this review, we contend that fulfilling the potential of brain substrate expansion will require a significant shift from current methods that emphasize direct manipulations of the brain (e.g., injections of cellular suspensions and the implantation of multi-electrode arrays) to the generation of more sophisticated neural tissues and neural-electric hybrids *in vitro* that are subsequently transplanted into the brain. Drawing from neural tissue engineering, stem cell biology, and neural interface technologies, this strategy makes greater use of the manifold techniques available in the laboratory to create biocompatible constructs that recapitulate brain architecture and thus are more easily recognized and utilized by brain networks.

## Introduction

Damage to the brain can have devastating effects on an individual’s life, ranging from the inability to interact with the world to the dissolution of the identity of self. While the potential of natural neuroplasticity mechanisms is often overlooked (Cramer et al., 2011; Chen et al., 2014), it is nonetheless a matter of fact that the regenerative capacity of the central nervous system (CNS) is limited, and recovery from disabling deficits is often incomplete. This problem has motivated widespread efforts to develop techniques for restoring neurological function, including independent voluntary movement and cognition.

Broadly speaking, restorative interventions for the damaged brain fall into two categories: (1) optimization of brain network performance; and (2) expansion of nervous system substrate. Examples of the former include plasticity-based therapies (Nahum et al., 2013), deep brain stimulation (Mayberg et al., 2005; Follett et al., 2010), cortical stimulation (Plautz et al., 2003), and emerging optogenetic approaches (Zhang et al., 2007; Liu et al., 2012). These therapies are likely to be most effective in relatively healthy brains, in which modulation of existing networks is adequate to produce the desired outcome (Schiff et al., 2007; Nouri and Cramer, 2011).

When the brain has incurred significant injury, however, it is unclear whether modulation of brain circuits alone can lead to acceptable levels of neurological recovery. Neural substrate expansion, in which new elements that can increase computational capacity are integrated into the host brain, is perhaps a more suitable therapeutic alternative in these more severe cases. Theoretically, this avenue of research holds immense potential for restoring and even enhancing brain function (Serruya and Kahana, 2008; Serruya, 2015), and the clinical translation of this concept could transform the prospects of patients suffering from congenital brain defects or acquired brain damage.

In recent years, both biological (Arvidsson et al., 2002; Gaillard et al., 2007; Niu et al., 2013; Michelsen et al., 2015) and electronic (Berger et al., 2011) substrates for repair have received attention. A common theme across the majority of these approaches is that they are performed *in situ* with respect to the brain, whether it is the injection of cells or viral vectors or the placement of electrode arrays. Directly interfacing with the brain is straightforward but has substantial limitations, including the inherent fragility of the brain and the restricted set of laboratory techniques that can be applied in the *in vivo* environment. We contend that future attempts to expand the substrate of the brain will need to make greater use of the *in vitro* setting in order to fully develop the exciting potential of this idea. Specifically, we hypothesize that substrate designed to incorporate features of normal brain structure will have the greatest chance of integrating into and restoring the function of brain networks. In this article, we will review the recent progress in the field of brain substrate expansion, define the obstacles hindering further gains, and explore how taking advantage of neural tissue engineering, stem cell biology, and neural interface techniques in the laboratory can accelerate progress in this arena.

## Theoretical Basis of Brain Substrate Expansion

Destruction of brain tissue impairs cerebral function by disrupting *computation* and *connectivity*. Damage to gray matter reduces the number of computational processing units, such as cortical columns, that are available for transforming convergent inputs into patterned divergent outputs while injury to gray and white matter disrupts connectivity, hindering the flow of information within and among computational centers. Both types of impairment, which often occur simultaneously, diminish the global computational capacity of the brain. It is generally the case that a larger spatial volume of brain damage correlates with worse recovery and poorer long-term outcome, although the degree of dysfunction also depends on the specific brain region where a given volume of tissue has been lost and individual heterogeneity factors that are not yet understood (Follett et al., 2009; Ius et al., 2011; Nouri and Cramer, 2011; Riley et al., 2011).

With these considerations in mind, one approach for restoring brain function after injury is to expand the neural substrate available to the brain for computation (Serruya and Kahana, 2008; Serruya, 2015). This strategy is based upon the assumption that the human brain is capable of recognizing additional substrate and incorporating it into the functioning of native networks. A variety of evidence suggests that this hypothesis could be true. After transplantation into injury cavities in the brain, fetal cortical grafts survive robustly and integrate with adjacent brain tissue (Girman and Golovina, 1990; Gaillard et al., 2007; Santos-Torres et al., 2009). These grafts can adopt appropriate brain function, such as receptive fields in visual cortex (Girman and Golovina, 1990). It has also been shown that an electronic implant can artificially connect different areas of the brain and induce stable changes in neuronal output (Jackson et al., 2006) and that cortical neuron cultures can form closed feedback loops with computer systems (Bakkum et al., 2008; Pizzi et al., 2009). Finally, cortical function can “spread” to areas of the brain whose own function has been vacated as a result of amputation (Elbert et al., 1994; Qi et al., 2000) or deafferentation (Pons et al., 1991). Such findings emphasize the inherent plasticity of the brain and the essential role it would play in any neural substrate expansion paradigm.

Two philosophies dominate the literature to explain how neural substrate expansion could improve brain function. Traditionally, brain function has been described in a modular manner, in which each anatomical region is linked to a particular function. Under this framework, neural substrate expansion would be predicated on the addition of discrete “brain modules” for specific purposes, much in the same way that computer processing cores can be dedicated to certain tasks. Recently, a more nuanced view has emerged that attributes brain function to spatially distributed and temporally dynamic neural networks (Bassett and Bullmore, 2009; Duffau, 2015). This connectionist understanding of the brain implies that neural substrate expansion strategies should focus as much on the restoration of the “edges” of the connectome (i.e., white matter connections) as on the “nodes” (i.e., gray matter computation centers). Defining strategies for restoring specific brain functions with new substrates requires an understanding of how the brain naturally reorganizes after injury, including patterns of adaptive and maladaptive plasticity. The extensive literature on post-injury plasticity mechanisms is beyond the scope of the current discussion but has been reviewed elsewhere (Keyvani and Schallert, 2002; Cramer et al., 2011; Nahum et al., 2013; Chen et al., 2014).

## Current Examples of Brain Substrate Expansion

Although not typically described in such terms, several neuro-restorative techniques currently under investigation fit the description of brain substrate expansion. Both biological and electronic approaches have been studied, almost all of which involve primarily direct manipulations of the brain. We will briefly review this work and discuss some of the obstacles that have impeded greater success and clinical translation.

### Biological Approaches

Cellular methods for expanding brain substrate have been studied for the past two decades using either endogenous or exogenous sources of neurons. Neurogenesis occurs in the adult mammalian brain in the subventricular zone (SVZ; Doetsch et al., 1999; Curtis et al., 2007; Sanai et al., 2011) subgranular zone of the hippocampal dentate gyrus (Eriksson et al., 1998), and perhaps other areas (Ernst et al., 2014; Feliciano et al., 2015). The generation of neurons in canonical sites of neurogenesis (i.e., the SVZ and dentate gyrus) is upregulated in the setting of brain insults, including traumatic brain injury (Yu et al., 2008; Thomsen et al., 2014) and stroke (Arvidsson et al., 2002), and it has been postulated that brain circuits could be repaired by capitalizing on this natural phenomenon. Recently, several groups have converted cerebral astrocytes into neurons *in vivo* via directed viral transduction (Niu et al., 2013; Magnusson et al., 2014), demonstrating the feasibility of transdifferentiation as a means of expanding the endogenous neuron pool. These induced neurons persist for several months in rodent models and integrate into local networks.

In terms of exogenous neuronal sources, the majority of transplantation studies have employed the injection of suspensions of neural lineage cells, including neural progenitors (Kelly et al., 2004; Jensen et al., 2013) as well as mature neurons (Czupryn et al., 2011; Weick et al., 2011). This approach inherently results in a disorganized arrangement of new neurons. Nonetheless, transplanted cells survive and functionally integrate into local host networks. With the introduction of induced pluripotent stem (iPS) cell-derived cortical neurons into the neonatal (Espuny-Camacho et al., 2013) and adult (Michelsen et al., 2015) mouse brain, long axons project to appropriate targets over time. Moreover, engrafted cells assume rudimentary cortical functions such as visual receptive fields in the adult brain (Michelsen et al., 2015).

Fewer studies have examined the transplantation of neural tissue with a pre-formed structural or network architecture. As discussed previously, fetal cortical grafts exhibit a significant degree of survival and host integration *in vivo* (Girman and Golovina, 1990; Gaillard et al., 2007; Santos-Torres et al., 2009), but ethical concerns have curtailed the pursuit of this technique as a translatable clinical therapy, especially in the United States. Only one example of transplantation of engineered neural tissue has been published thus far. Small networks of hippocampal neurons grown on colloidal beads *in vitro* were delivered into the hippocampus of young adult rats (Jgamadze et al., 2012). The transplanted neurons migrated away from the beads and dispersed throughout the hippocampus, extending processes that formed functional connections with the host.

Despite the successes outlined above, several factors have limited additional progress in developing biological approaches to brain substrate expansion. Stem cells are better able to withstand the hostile environment of damaged brain than differentiated neurons, but it is difficult to direct the differentiation of neural precursors, either endogenous or exogenous, into the desired neuronal sub-types *in vivo*. While current substrate expansion approaches result in significant numbers of neurons, they may not provide adequate biomass and support cells (e.g., glia and endothelial cells) to reconstitute large human brain defects. Beyond matters of neuron numbers, the functional significance of these new neurons is unclear. There are abundant examples of new neuron integration with local circuits. However, evidence that this neural substrate contributes to the function of large-scale native networks is sparse (Czupryn et al., 2011; Michelsen et al., 2015). Although this deficiency could be the result of a lack of experimental emphasis or suitable models, it may be that these biological strategies for neural substrate expansion lack the fundamental organization to support complex brain function. The highly precise architecture of the brain, at the scale of microscopic structure and network-level connections, dictates the function of the brain in both the healthy and diseased states (Bassett and Bullmore, 2009; Stoner et al., 2014). For example, cortical columns, whose function relies upon the precise connectional relationships among neurons in different layers, are thought to drive computational activity within the cerebral cortex (Buxhoeveden and Casanova, 2002; Meyer et al., 2013). Other than fetal cortical grafts, all of the biological approaches are characterized by a relatively random distribution of new neural elements within the brain. Moreover, the axonal projections of new neurons may be too few to support the degree of reconnection among different brain regions necessary to restore function.

### Electronic Approaches

In the realm of man-made devices, true examples of brain substrate expansion are more limited. These neural interfaces fall into the broad categories of devices that perform computational tasks and relay systems that facilitate communication between spatially distinct neural tissues. A second class of interfaces promotes the ability of the brain to interact with the external world. These devices, which include sensory modality replacements and conventional brain-machine interfaces (BMIs), are not designed to restore the function of a damaged brain; in fact, they typically require an intact brain for optimal usage. Nevertheless, they share similar technologies and obstacles with electronic brain substrates and thus will also be briefly surveyed.

The hippocampal prosthetic pioneered by Deadwyler, Hampson, and Berger is a well-documented example of an electronic substrate that seeks to reproduce cerebral computational activity. Using a multi-input/multi-output nonlinear model to predict hippocampal outputs based on monitored inputs, a device simulates basic hippocampal function by delivering appropriate stimulation to hippocampal output regions (Berger et al., 2011). This interface appears to improve memory encoding in an intact animal as well as recover of memory function when the native hippocampus has been compromised by pharmacological inhibition of synaptic transmission. Similar prosthetics have been constructed to simulate prefrontal cortex (Hampson et al., 2012) and cerebellar activity (Herreros et al., 2014). While these studies are promising proofs of concept, they are based upon experimental tasks that may not generalize to natural animal behavior and often do not have a direct human behavioral correlate. Electronic devices emulating neural circuits that produce naturalistic human behavior and cognition have not yet been created.

Other interface systems have replicated the function of axonal pathways. In a non-human primate model, an electronic neural implant capable of autonomous recording and stimulation enabled the synchronization of neural activity from two discrete cortical regions, resulting in the reorganization of motor output representations (Jackson et al., 2006). This implant enabled the transmission of information between different brain structures and supported network computational activity, the essential functions of white matter tracts. The same relay strategy has been adapted to connect the brain directly to muscles in the upper extremity as a strategy to bypass spinal cord or peripheral nerve injuries (Moritz et al., 2008). These studies recreated naturally existing nervous system pathways, but more recent investigations have sought to develop artificial neural connections. Patterns of sensorimotor (Pais-Vieira et al., 2013) and memory (Deadwyler et al., 2013) information recorded from the brains of trained “donor” animals have been transferred to naïve “recipient” animals via electrical stimulation, leading to improved performance on behavioral tasks in the absence of training in the recipient animals. Similarly, several groups have raised the possibility of transferring data between human test subjects using non-invasive modalities such as scalp electroencephalography (EEG) and transcranial magnetic stimulation (TMS; Grau et al., 2014; Rao et al., 2014). In these brain-to-brain interfaces, it has been suggested that healthy brains could serve as computational substrate and data sources for damaged brains or that complex tasks could be performed using the summed computational capacities of multiple brains (Pais-Vieira et al., 2015).

With regards to sensory modality replacements, the most clinically successful neural substitute to date has been the cochlear implant. Auditory stimuli is captured and processed by an external receiver, which then delivers stimulation to spiral ganglion neurons in a tonotopic manner via an intracochlear electrode array (Carlson et al., 2012). In this way, hearing is restored sufficiently to enable speech discrimination. However, significant perceptual challenges remain, including problems discerning speech in a noisy environment, impaired pitch perception, and poor sound localization. Optical neural stimulation, implants within the cochlear nerve itself, and pharmacological preservation of neurons adjacent to the implant may enable next-generation devices to overcome these barriers (Roche and Hansen, 2015). Thematically similar efforts have been pursued to restore sight using either retinal implants (Zaghloul and Boahen, 2006; Chader et al., 2009; Ho et al., 2015) or cortical visual prostheses (Lewis et al., 2015).

As opposed to restoring inputs into the brain, conventional BMIs focus on reestablishing the output functions of the brain. Two teams have demonstrated the feasibility of driving robotic devices using decoded brain activity in quadriplegic patients (Hochberg et al., 2012; Collinger et al., 2013). Similar systems have enabled patients to control the movement of cursors on a computer screen and type messages (Gilja et al., 2015; Jarosiewicz et al., 2015). These studies required the implantation of high-density arrays of penetrating microelectrodes into the brain to record the activity of multiple individual neurons. The translational potential of methodologies that record local field potentials such as electrocorticography (ECoG; Schalk et al., 2008; Wang et al., 2013) and EEG (Chaudhary et al., 2015) also is being actively investigated.

Across all electronic approaches for restoring neurological function, a prerequisite for a high-performance device is a stable neural interface. The lack of such a stable interface has been recognized as a key impediment to the long-term utility of neural interface technologies, especially for those methods based on unit recordings (Judy, 2012). Because of the mismatch in material properties between standard metal and silicone electrodes and the brain, micro-motion at the interface causes shifts in the ensemble of neurons available for recording and promotes glial scarring, which reduces the signal-to-noise ratio of recorded neural activity. Various techniques have been proposed to solve these problems, including improving the biocompatibility of electrodes (Marin and Fernández, 2010; Fattahi et al., 2014) and the development of “softer” flexible electrode arrays (Viventi et al., 2011; Kim et al., 2012; Kuzum et al., 2014). While these approaches could ultimately minimize glial scarring, they are unlikely to eliminate the need for frequent system recalibrations by trained technicians to mitigate the effects of shifting neural waveforms. Chronically implanted electrode arrays also demonstrate impedance losses unrelated to inflammation or gliosis that compromise the ability to record single unit activity. ECoG strategies based on local field potentials rather than unit activity are not beholden to changes in individual neural waveforms over time, but they may provide less neural information overall.

## Exploiting the *In Vitro* Setting for Future Gains

For the current substrate expansion approaches that have been explored, a common refrain has been an emphasis on direct manipulations of the brain. Thus, biological methods have relied upon cerebral injections of cell suspensions or viral vectors while electronic strategies have been predicated upon the implantation of multi-electrode arrays in the brain. *In vivo* work with the brain is inherently limited by the fragility of cerebral tissue and the lack of modularity of intact brain (i.e., the brain cannot be taken apart and put back together without significant consequences). We contend that these constraints have greatly hindered the utilization of brain substrate expansion for neurological restoration.

As opposed to the *in vivo* setting, the *in vitro* environment offers access to a broader set of experimental techniques and an improved ability to measure and control outcomes (Cullen et al., 2011a,b). For example, specific neuron sub-types can be differentiated from stem cell sources, genes can be overexpressed with minimal concern for contamination of adjacent brain structures, and neurons can be more easily stimulated and recorded using a variety of modalities. Thus far, the *in vitro* setting has only rarely been utilized for the purposes of brain repair (Jgamadze et al., 2012). We postulate that the flexibility of this approach will be essential for accelerating progress in the field of brain substrate expansion. Actuating this paradigm shift will involve creating synergies between tissue engineering, stem cell biology, and novel neural interface technologies. Although it is true that constructs created in the laboratory will ultimately need to be brought into the *in vivo* environment, we hypothesize that these engineered structures will be better equipped to integrate with the brain, provide more stable input/output interfaces, and reconstruct high-level brain circuitry. In remainder of this review, we will describe some intriguing possibilities of using the *in vitro* setting to expand brain substrate and the keys to their successful implementation.

### Engineering Neural Tissue with Brain-Specific Architecture

Brain function is intimately related to brain architecture. As evidenced in patients with autism, even subtle disorganization of brain microanatomy may result in network and behavioral dysfunction (Stoner et al., 2014). Thus, in thinking about generating new tissue-based neural substrate *in vitro*, an important consideration may be creating cytoarchitecture that is recognizable to the brain and capable of carrying out computational activity. There are several potential avenues to achieving this objective, both for the computational centers of the brain and the connections between these centers.

Prior attempts at engineering neural tissue have been limited primarily to dissociated neuronal cultures grown in three-dimensional (3D) hydrogels (O’Connor et al., 2001; Tian et al., 2005; Ju et al., 2007; Irons et al., 2008; Xu et al., 2009). These constructs were relatively fragile and lacked any true brain-specific structure. Several methodologies have since been developed that could facilitate the creation of more complex tissue architectures. Silk sponges support robust cortical neuron growth and can be assembled into layered arrangements (Tang-Schomer et al., 2014b). Alternatively, 3D printing techniques can deposit layers of neurons on a surface in a highly controlled manner (Lozano et al., 2015). Decellularization processes to isolate the extracellular matrix scaffold of the brain also may be useful in reconstructing particular brain structures (Baiguera et al., 2014). A common obstacle to all of these approaches is seeding the correct neuronal subtype within a given part of the tissue. It is unclear whether sufficient cues could be embedded in engineered neural tissue to permit appropriate neuronal migration patterns.

Beyond serving as sources of autologous neurons for tissue engineering applications, stem cells provide another route for expanding brain substrate via their property of self-aggregation. Given the right environmental cues, embryonic stem (ES) and iPS cells form organ-like tissues that recapitulate developmental processes (Sasai, 2013). The resultant structures exhibit an incredible level of organ-specific architectural detail. In the realm of brain organogenesis, an early example of this self-organizing property was the growth of an optic cup structure that possessed retinal morphology (Eiraku et al., 2011). A subsequent study created cerebral organoids that formed several discrete brain regions, including the cerebral hemispheres, hippocampus, ventricular system, and choroid plexus (Lancaster et al., 2013). Other studies have emphasized self-organizing models of the cerebral cortex and demonstrated rudimentary specification of cortical layers (Kadoshima et al., 2013; Pasca et al., 2015). From the regenerative perspective, two areas that could benefit from further investigation are additional protocols for generating specific brain structures (e.g., hippocampus, basal ganglia, etc.) and methods for maturing these organoids into more functional tissue.

In comparison to restoring neuronal cytoarchitecture, less attention has been paid to the reconstruction of axonal networks within the brain. However, with a growing emphasis on connectivity as an essential determinant of brain function, it could be argued that white matter substrate is just as, if not more, important as gray matter substrate for expanding brain circuitry. The barriers to axonal regeneration *in vivo*, including inherently slow axon growth rates and the harsh environment of the injured brain, are well documented (Yiu and He, 2006; Liu et al., 2011). The promise of an *in vitro* laboratory approach to axon growth has been demonstrated in a group of pioneering studies, in which mechanical forces produced dense tracts of “stretched” axons (Smith et al., 2001; Pfister et al., 2004; Smith, 2009). Exploiting a mechanism wholly separate from growth cone-mediated axonogenesis, stretch axon growth has yielded tracts up to 10 cm in length using dorsal root ganglia neurons (Pfister et al., 2004). This axonal tissue has been utilized to repair animal models of spinal cord (Iwata et al., 2006) and peripheral nerve injuries (Huang et al., 2009). Stretch axon growth also has been applied to cerebral neuron subtypes (Smith et al., 2001), suggesting the applicability of this methodology to the brain. For circuitry on a smaller scale, the conceptually similar method of growing axon tracts within micron-scale hydrogel conduits has been developed (Cullen et al., 2012). Ultimately, combining this technology with axon stretch will create a comprehensive suite of axonal constructs that can address a broad range of disconnection disorders in the brain.

Improvements to the scaffolding around engineered tracts are needed in order to provide physical support for delicate cerebral axons and the means to easily manipulate the tissue constructs during surgical implantation. Careful characterization of the integrity and capacity for information transfer of these axon tracts *in vitro* also will be necessary prior to *in vivo* transplantation studies. Finally, data transfer across engineered and native axons *in vivo* will require comparison to establish how effectively the former can contribute to the normal function of neuronal networks and support oscillations and other complex spatio-temporal coding strategies.

### “Brain Modules” Incorporating Stable Neural Interfaces

As discussed previously, one of the central obstacles facing invasive interfaces with the brain is interface stability. We posit that the *in vitro* setting provides a potential route for mitigating this problem. It is well documented that neurons can be cultured on a variety of surfaces, including not only the plastics used in conventional tissue culture plates but also organic polymers (Cullen et al., 2008) and metals (Hales et al., 2010). These observations suggest that it might be possible to integrate multi-electrode arrays and other electronic devices within engineered neural tissues, similar to the ones described in the prior section. Such hybrid constructs could then be transplanted into the brain, relying on the formation of functional synapses between the engrafted tissue and the brain for communication with electronic systems (Cullen et al., 2011a; Cullen and Smith, 2013). Depending on the format of the engineered tissue, access to both superficial and deep targets in the brain could be obtained.

With electrodes embedded directly into engineered neural tissue, it is less likely that shifting will occur between neurons and electrode contacts. Thus, inflammation and glial scarring may occur less frequently while the stability and fidelity of neuronal recordings could be improved. Although it is possible that transplanted neurons could migrate away from the electrodes or tissue scaffold *in vivo*, we hypothesize that such events would be infrequent if the neurons have already formed a robust neuronal network *in vitro* and stable new extracellular millieu. In other words, the neurons would be “trapped” in proximity to the electrodes by the physical constraints of being part of a tissue and the network constraints of having formed numerous synaptic connections with neighboring cells. One other potential concern regarding interface stability is tension resulting from wire tethering. Obviously, wireless telemetric systems, such as the “neural dust” concept (Seo et al., 2015), would obviate this issue. While these technologies are being developed and tested, flexible electronics that conform to the topography of the brain could serve as an alternative solution (Kim et al., 2010, 2012).

Perhaps the first example of a hybrid interface was the neurotrophic electrode designed by Philip Kennedy (Kennedy et al., 1992; Bartels et al., 2008). These glass cone electrodes contain a neurotrophic medium that promotes the ingrowth of neurites and other tissue elements. Electrical signals from the neurites are then transmitted to wires within the cone electrode. In this methodology, the electrode gradually becomes embedded in the neuropil itself, which promotes a more stable interface. Other early studies have more directly explored the concept of integrating electrodes with neurons prior to implantation. Neurons have been grown on conducting polymer fibers (Cullen et al., 2008) and silk-based flexible electrodes (Tang-Schomer et al., 2014a) in *in vitro* in formats amenable for transplantation. Although no *in vivo* studies have been performed thus far, this work suggests the feasibility of constructing neural-electric hybrids.

Moving forward, one of the main challenges will be designing scaffold architectures that promote the growth of 3D neuronal networks on and around the active components of the integrated electronics. These designs will draw upon expertise in the fields of tissue and electrical engineering. Devising methods for physically manipulating the hybrid constructs without inducing neuronal damage will be necessary. Reliable flexible electronics that provide consistent long-term functionality will be equally important.

## Conclusion

One of the primary objectives of modern neuroscience research is the restoration of neurological and cognitive function after insults to the brain. Expanding the substrate for cerebral computation, either through biological or electronic methods, has vast potential for helping to achieve this goal. Thus far, this strategy has been limited because it has emphasized direct manipulations of the brain, such as injections of cellular suspensions or viral vectors and the implantation of electrode arrays. We believe that the *in vitro* setting can promote progress in reconstructing brain circuitry by providing a host of tools that is relevant to tackling obstacles encountered in current substrate expansion strategies. As *in vitro* substrate expansion methodologies are explored more fully, we predict that previously separate domains will increasingly merge. Neural-electric hybrids will become more common, bringing together the fields of cell- and tissue-based repair and neural interface technologies, and brain substrate expansion itself will blend with conventional techniques for brain circuit modulation. The quest for functional brain restoration is one in its early infancy. However, embracing the concept of generating more complex neural substrates in the laboratory prior to their introduction into the brain will accelerate the maturation of this field.

## Author Contributions

HIC and DJ drafted the manuscript. MDS, DKC, JAW, and DHS provided critical review of the manuscript and provided specific input in their respective areas of expertise.

## Conflict of Interest Statement

The authors declare that the research was conducted in the absence of any commercial or financial relationships that could be construed as a potential conflict of interest.
